# Microbiota attenuates chicken transmission-exacerbated campylobacteriosis in *Il10*^*−/−*^ mice

**DOI:** 10.1038/s41598-020-77789-2

**Published:** 2020-11-30

**Authors:** Ying Fu, Ayidh Almansour, Mohit Bansal, Tahrir Alenezi, Bilal Alrubaye, Hong Wang, Xiaolun Sun

**Affiliations:** 1 CEMB, University of Arkansas, Fayetteville, AR 72701 USA; 2 Center of Excellence for Poultry Science, University of Arkansas, 1260 W Maple St. O-409, Fayetteville, AR 72701 USA

**Keywords:** Infection, Bacterial infection, Colonic diseases

## Abstract

*Campylobacter jejuni* is a prevalent foodborne pathogen mainly transmitting through poultry. It remains unknown how chicken-transmitted *C. jejuni* and microbiota impact on human campylobacteriosis. *Campylobacter jejuni* AR101 (Cj-P0) was introduced to chickens and isolated as passage 1 (Cj-P1). *Campylobacter jejuni* Cj-P1-DCA-Anaero was isolated from Cj-P0-infected birds transplanted with DCA-modulated anaerobic microbiota. Specific pathogen free *Il10*^*−/−*^ mice were gavaged with antibiotic clindamycin and then infected with Cj-P0, Cj-P1, or Cj-P1-DCA-Anaero, respectively. After 8 days post infection, *Il10*^*−/−*^ mice infected with Cj-P1 demonstrated severe morbidity and bloody diarrhea and the experiment had to be terminated. Cj-P1 induced more severe histopathology compared to Cj-P0, suggesting that chicken transmission increased *C. jejuni* virulence. Importantly, mice infected with Cj-P1-DCA-Anaero showed attenuation of intestinal inflammation compared to Cj-P1. At the cellular level, Cj-P1 induced more *C. jejuni* invasion and neutrophil infiltration into the *Il10*^*−/−*^ mouse colon tissue compared to Cj-P0, which was attenuated with Cj-P1-DCA-Anaero. At the molecular level, Cj-P1 induced elevated inflammatory mediator mRNA accumulation of *Il17a*, *Il1β*, and *Cxcl1* in the colon compared to Cj-P0, while Cj-P1-DCA-Anaero showed reduction of the inflammatory gene expression. In conclusion, our data suggest that DCA-modulated anaerobes attenuate chicken-transmitted campylobacteriosis in mice and it is important to control the elevation of *C. jejuni* virulence during chicken transmission process.

## Introduction

*Campylobacter jejuni* asymptomatically colonizes chicken gut, but it is one of the prevalent foodborne pathogens in the developed countries. Chicken is the main vector to transmit *C. jejuni* to host humans, and the bacterium was detected in 20–56% of chicken breast meat from various brands in 2013^1^. More than 12.2 campylobacteriosis cases per 100,000 population were recorded in 2017 in the USA. That number represented a 4.3% increase compared to 2016 and was higher than the combined incidences by the following 8 bacterial pathogens^2^. A total of 1.3 million individuals are afflicted by the disease, resulting in 76 deaths every year^3^. Furthermore, *C. jejuni* infection causes severe post-infectious complications, including arthritis^4^, the neurodegenerative disorder Guillain-Barré Syndrome^5^, Irritable Bowel Syndrome^6^, and Inflammatory Bowel Diseases (IBD)^7,8^.

Although reducing chicken meat *Campylobacter* counts by 2 logs is estimated to decrease a 30-fold in human campylobacteriosis^9^, current strategies to reduce campylobacteriosis are not effective enough^10^. This was evidenced by the relative consistent rate of campylobacteriosis incidences in the Morbidity and Mortality Weekly Report at CDC infectious disease database from January, 1996 to August, 2016^11^. Interestingly, it has been largely overlooked whether any important events happen when *C. jejuni* is transmitted from vector chickens to host humans. Foodborne bacterial pathogen contamination is mainly determined by enumerating the pathogens using bacterial culture or PCR method without assessing the possibility of bacterial infection ability alteration after the bacterial animal colonization^12^. *Campylobacter jejuni* is present in chicken farms for many years, and the bacterium is transmitted through many batches of chickens^13^. Consequently, it remains elusive whether *C. jejuni* chicken transmission impacts its subsequent colonization and campylobacteriosis induction.

*Il10*^*−/−*^ mouse infection model has recently been successfully developed to mimic human campylobacteriosis in various labs^14–19^. Specific pathogen free (SPF) *Il10*^*−/−*^ mice resisted against *C. jejuni*-induced colitis, while the mice were susceptible to campylobacteriosis after treated with anaerobe-killing antibiotic clindamycin^20^. Using HPLC/MS analysis, we found that clindamycin depleted all secondary bile acids, particularly deoxycholic acid (DCA)^20^. Furthermore, anaerobe metabolite, DCA, was able to prevent and treat *C. jejuni*-induced colitis in ex-GF mice^20^. We also found that DCA resisted against chicken colonization of *C. jejuni* human clinical isolate 81–176 and chicken isolate AR101^21^. The microbiota composition at the cecal of the infected birds transplanted with DCA-modulated microbiota was assessed at phylum level using real-time PCR, and the results showed that the microbiota compositions were different^21^. *Campylobacter jejuni* motility and adherence to cells aren’t changed in the presence of DCA, although DCA induced virulence genes *cia*B, *cme*ABC, *dcc*R, and *tly*A^22^. However, it is unclear whether DCA regulates *C. jejuni* chicken transmission and subsequent induction of campylobacteriosis.

In this study, we found that chicken-transmitted *C. jejuni* (passage 1, Cj-P1) induced more severe intestinal inflammation in *Il10*^*−/−*^ mice compared to the non-transmitted bacterium (passage 0, Cj-P0), while Cj-P1-DCA-Anaero induced less colitis, bacterial invasion, and inflammatory gene expression in *Il10*^*−/−*^ mice compared to Cj-P1. Thus, *C. jejuni* transmitted from birds raised in various conditions could behave differently in inducing enteritis. The outcome of this study will provide key information about the interplay between chicken microbiome, *C. jejuni* transmission, and host susceptibility and response, and could help the development of new prevention strategies against foodborne pathogens.

## Material and methods

### Campylobacter jejuni strains isolated from infected birds

*Campylobacter jejuni* strain AR101 (Cj-P0) was isolated from experimental chickens at Dr. Billy Hargis’s laboratory at the University of Arkansas at Fayetteville and the bacterium was used in our recent report^21^. In the report, *C. jejuni* in the cecal digesta of the 28 days of age birds infected with Cj-P0 was cultured on *C. jejuni* selective blood plates with five antibiotics (cefoperazone, cycloheximide, trimethoprim, vancomycin and polymyxin B) for 48 h at 42 °C using the GasPak system (BD), and the isolated *C. jejuni* was named as Cj-P1 (*C. jejuni* passage 1). In the report, *C. jejuni* in the cecal digesta of 28 days of age birds fed DCA and infected with Cj-P0 was cultured on the selective *C. jejuni* plates and was named as Cj-P1-DCA. Cecal digesta from uninfected 28 days of age birds fed with DCA diet was collected and cultured on Brain Heart Infusion (BHI) plates under anaerobic or aerobic conditions, and the isolated bacteria were named anaerobic-microbiota (DCA-Anaero) or aerobic-microbiota (DCA-Aero), respectively. *Campylobacter jejuni* in cecal digesta of 28 days of age birds colonized with DCA-Anaero or DCA-Aero and infected with Cj-P0 was cultured on the selective *C. jejuni* plates and was named as Cj-P1-DCA-Anaero or Cj-P1-DCA-Aero, respectively. The microbiota at the cecal of those birds was assessed at phylum level using real-time PCR, and the results showed that the microbiota compositions were different^21^. The AR101 strains were routinely grown on the selective *C. jejuni* plates and examined under microscopy for size, morphology and motility”.

### Campylobacter jejuni motility assay

Cj-P0, Cj-P1, Cj-P1- DCA, Cj-P1-DCA-Anaero or Cj-P1-DCA-Aero was grown on the selective plates, collected, and diluted to an optical density at 600 nm (OD_600_) of 1. Each bacterium of 1 μl was then stabbed into a 0.4% agar Brain Heart Infusion (BHI) plate without antibiotic cocktail. The less dense agar facilitated *C. jejuni* to easier move inside the agar and the bacterium formed a halo of growth around the inoculation point. Following microaerobic growth at 42 °C for 24 h, the radius of the ring was calculated relative to that of Cj-P0. Cj-P0 was grown on each plate to control plate-to-plate variation. Experiments were performed in triplicate and repeated three times.

### Mouse experiment

Animal experiments were performed in accordance with the Animal Research: Reporting of In Vivo Experiments (https://www.nc3rs.org.uk/arrive-guidelines). The experiments were approved by the Institutional Animal Care and Use Committee of the University of Arkansas. For *C. jejuni* infection experiments, cohorts of 5 to 9 SPF C57BL/6 *Il10 *^*−/−*^ mice were orally gavaged daily with antibiotic clindamycin (Sigma-Aldrich) at 67 mg/kg body weight (BW) for 7 days. One day after the last antibiotic treatment, the mice were gavaged with a single dose (10^9^ CFU/mouse) of Cj-P0 (5 mice), Cj-P1 (9 mice), Cj-P1-DCA-Anaero (5 mice), Cj-P1-DCA (9 mice), Cj-P1-DCA-Aero (5 mice), respectively as described above. Mice were followed clinically for evidence of diarrhea, failure to thrive, and mortality. At the end of experiments at 8 days post infection, tissue and stool samples from mouse colon were collected for protein, RNA, histology, and culture assay. For live *C. jejuni* counting, MLN and spleen were aseptically resected. Colon tissue was opened, resected, and washed three times in sterile PBS. Colonic luminal content (stool) was also collected. The freshly collected tissues and stool were weighed, homogenized in PBS, serially diluted, and cultured on selective *C. jejuni* plates supplemented with 5 antibiotics cocktail (cefoperazone, cycloheximide, trimethoprim, vancomycin, and polymyxin B) for 48 h at 37 °C using the GasPak system (BD Biosciences) as described before^21^. *Campylobacter jejuni* colonies were counted, and data were presented as CFU per gram tissue or stool. Histopathological images were acquired using a Nikon TS2 fluorescent microscope^23^. Intestinal inflammation was scored using a scoring system from 0–4 as showed before^20,24^.

### Fluorescence in situ hybridization (FISH)

*C. jejuni* at intestinal tissue sections was visualized using FISH assay as previously described^24^. Briefly, tissue sections were deparaffinized and hybridized with the FISH probe for up to 48 h. The tissue sections were then washed, and the mammalian nuclei were visualized by staining with DAPI. The tissue sections were then imaged using the Nikon TS2 fluorescent Microscope system.

### Immunohistochemistry (IHC)

Neutrophils in intestinal tissues were detected using anti-myeloperoxidase (MPO) IHC analysis as described previously^25^. Briefly, intestinal tissue sections were deparaffinized, blocked, and incubated with an anti-MPO antibody (1:400; Thermo Scientific) overnight at 4 °C. After incubation with anti-rabbit biotinylated antibody and three times washing, the tissue sections were incubated with avidin/biotin complex (Vectastain ABC Elite Kit, Vector Laboratories). After three times washing, the tissue sections were added with diaminobenzidine (Dako) within 2 min. The mammalian nuclei were visualized by staining with hematoxylin (Fisher Scientific). The tissue sections were then imaged using the Nikon TS2 Microscope system.

### Real-time RT-PCR

Total RNA from colonic tissue was extracted using TRIzol (Invitrogen). cDNA was prepared using M-MLV (Invitrogen). mRNA levels of proinflammatory genes were determined using the SYBR Green PCR Master Mix (Bio-Rad) on a Bio-Rad 384-well Real-Time PCR System and normalized to *Gapdh.* The primer sequences of the genes *Gapdh*, *17a*, *Il1β*, and *Cxcl1* were reported before^20^.

### White blood cell isolation and migration assay

Blood was collected from *Il10*^*−/−*^ mice and the red blood cells were lysed in the buffer of 8.3 g/l NH_4_Cl in 0.01 M Tris–HCl buffer of pH 7.5. The collected white blood cells were resuspended in 1% FBS RPMI 1640 medium. Cells were plated at 10^4^ per insert in 24‐well Transwells (Corning) with 3 μm pores and incubated at 37 °C and 5% CO_2_. Cj-P0, Cj-P1, Cj-P1-DCA, Cj-P1-DCA-Aero, and Cj-P1-DCA-Anaero at 10^5^ CFU/well were inoculated into the bottom wells. White blood cells migrated into the bottom well were imaged and counted one hour later using the Nikon TS2 Microscope system ,similar to previously described^25^. Cells in six fields per well were counted.

### Statistical analysis

Values were displayed as mean ± standard error of the mean as reported before^20^. Differences between groups were analyzed using the nonparametric Mann–Whitney *U* test with Prism 7.0 software. Experiments were considered statistically significant if *P* value was < 0.05.

### Ethics approval and consent to participate

All animal protocols were approved by the Institutional Animal Care and Use Committee of the University of Arkansas at Fayetteville.

## Results

### Chicken-transmitted C. jejuni induced more severe intestinal inflammation

**Figure 1:**
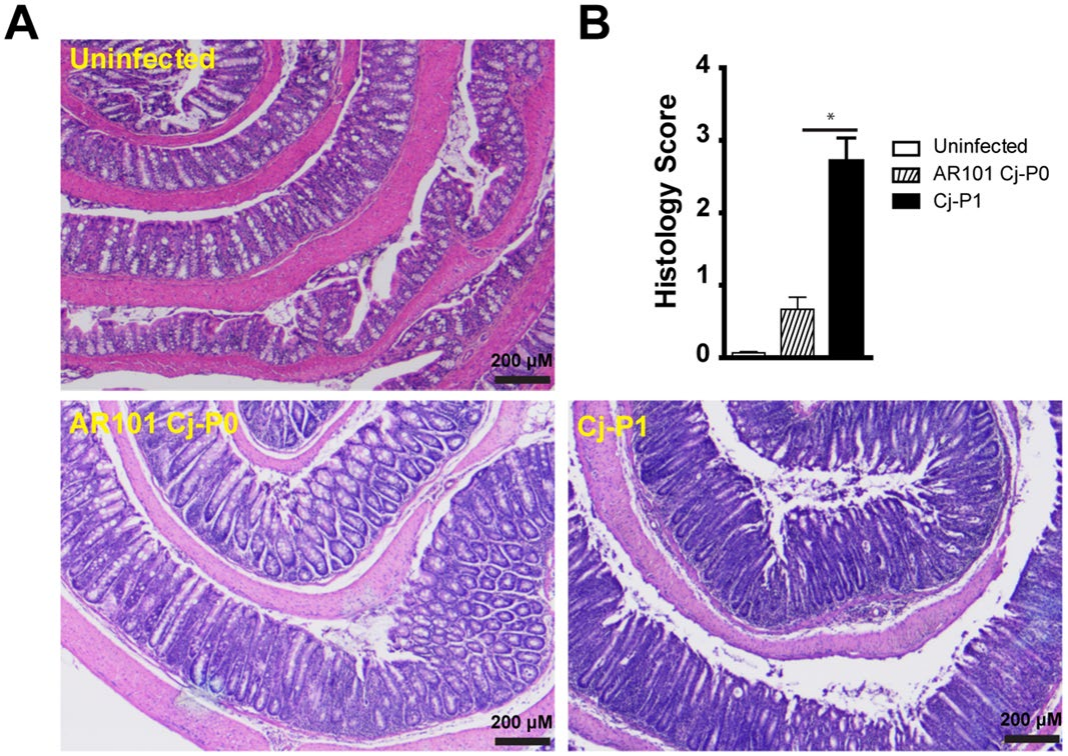
Chicken-transmitted *C. jejuni* (Cj-P1) induced more severe colitis compared to AR101 (Cj-P0). Cohorts of 5–9 SPF *Il10*^*−/−*^ mice were orally gavaged daily with clindamycin for 7 days. The mice were then gavaged with a single dose of 10^9^ CFU/mouse *C. jejuni* of Cj-P0 (5 mice) and Cj-P1 (9 mice) and were euthanized 8 days post-infection. (**A**) H&E staining showing representative intestinal histology of *C. jejuni*-induced colitis in *Il10*^*−/−*^ mice. (**B**) Quantification of histological intestinal damage score. *, P < 0.05. Data represent means ± SEM. Scale bar is 200 μm. Results are representative of 3 independent experiments.

To address whether the asymptomatic *C. jejuni* transmission in chickens influenced its induction of intestinal inflammation in susceptible hosts such as human or *Il10*^*−/−*^ mice, we cultured infected bird cecal content, isolated *C. jejuni*, and labeled it as passage 1 or Cj-P1. We reasoned that *C. jejuni* transmitting through chickens altered virulence and would induce worse intestinal inflammation. To examine this hypothesis, SPF *Il10*^*−/−*^ mice were orally gavaged daily with antibiotic clindamycin for 7 days. The mice were then infected with Cj-P0 or Cj-P1 with a single oral gavage dose of 10^9^ CFU/mouse. Interestingly, after 6 days post-infection, *Il10*^*−/−*^ mice infected with chicken-transmitted Cj-P1 showed clinical sign of enteritis. After 8 days post infection, *Il10*^*−/−*^ mice infected with Cj-P1 demonstrated severe morbidity and bloody diarrhea and the experiment had to be terminated. At cellular level, Cj-P0 induced mild intestinal inflammation in the *Il10*^*−/−*^ mice, shown as crypt hyperplasia, mild goblet cell depletion, and mild immune cell infiltration into lamina propria (Fig. 1A). Remarkably, Cj-P1 induced more severe intestinal inflammation and higher histopathological score compared to Cj-P0 ( 2.8 vs. 0.8, P < 0.05) , shown as crypt abscesses, extensive immune cell infiltration and massive goblet depletion (Fig. 1A,B). These results suggest that *C. jejuni* transmission through chickens enhances infection capacity and induces more severe intestinal inflammation.

### Chicken-transmitted Cj-P1 aggressively invaded colon tissue

**Figure 2:**
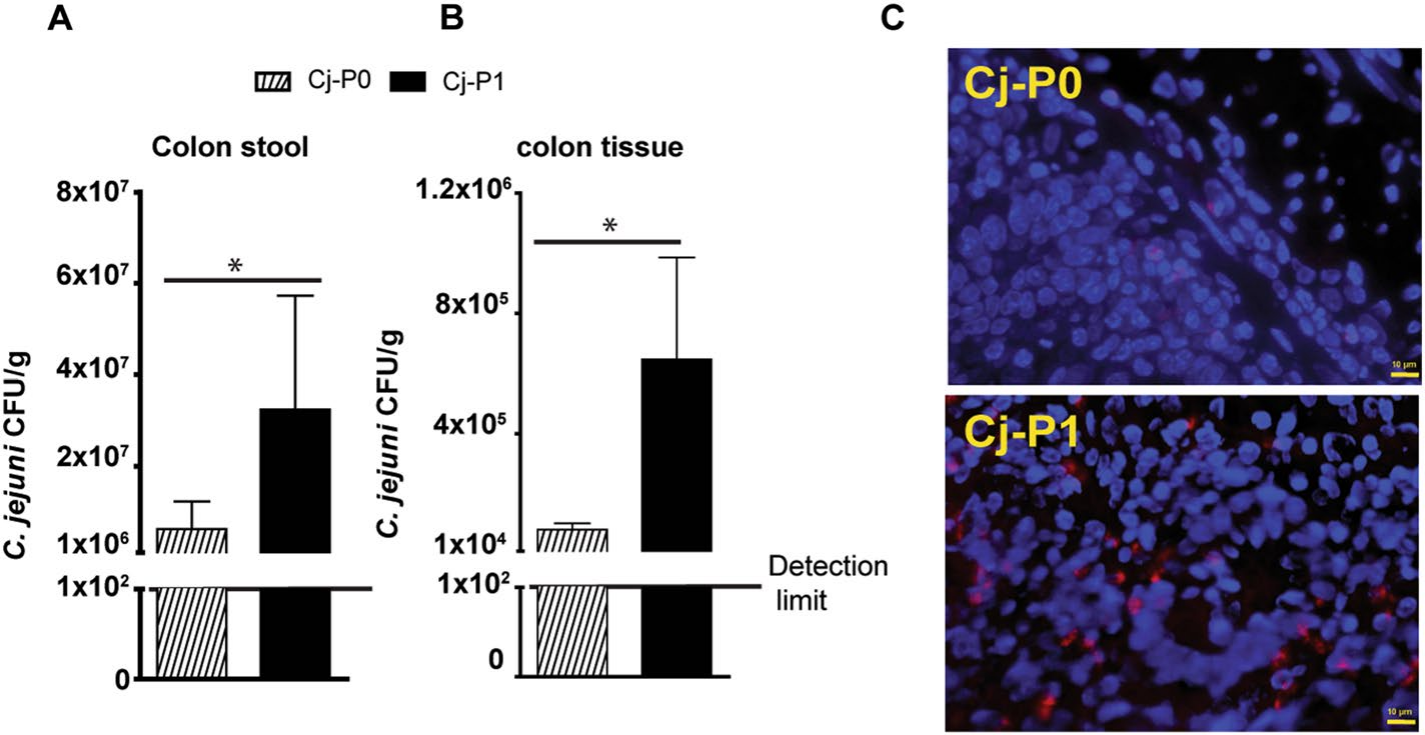
Cj-P1 aggressively invaded into colon tissue. Cohorts of mice were infected as in Fig. 1. Samples from colon content and tissue were collected and aseptically cultured. (**A**) *C. jejuni* count in colon content. (**B**) *C. jejuni* count in colon tissue. (**C**) Clustered *C. jejuni* (red dots) in colonic section of infected mice was detected using FISH. Scale bar is 10 μm. *, P < 0.05. Data represent means ± SEM. Results are representative of 3 independent experiments.

*Campylobacter jejuni* colonization and invasion are essential for its successful induction of campylobacteriosis^26^. To investigate the mechanism of how Cj-P1 induced more intestinal inflammation compared to Cj-P0, we next evaluated *C. jejuni* colonization and invasion in colon. Colon content and tissue were weighed, homogenized, serially diluted, and cultured on selective plates. Notably, the luminal colonization level of *C. jejuni* Cj-P1 was significantly denser in mouse colon compared to that of mice infected with Cj-P0 (3.24 × 10^7^ vs. 6.22 × 10^6^ CFU/g stool, P = 0.049) (Fig. 2A). We then examined *C. jejuni* invasion into colon tissue. In consistent with luminal *C. jejuni* colonization levels, Cj-P1 significantly invaded into colon tissue compared to Cj-P0 (6.47 × 10^5^ vs. 8.12 × 10^4^ CFU/g tissue, P = 0.016) (Fig. 2B). To further detect the *C. jejuni* spatial distribution in colon tissue, we visualized *C. jejuni* DNA using fluorescence in situ hybridization (FISH) and fluorescence microscopy imaging. Notably, while Cj-P1 DNA was detected widely in the inflamed crypts and the lamina propria section of the mouse intestine, Cj-P0 was seldomly detectable in the mouse colon (Fig. 2C). These results indicate that Cj-P1 gains virulence ability to invade more aggressively into colonic crypts.

### Transmitted Cj-P1 induced severe crypt abscesses in colon

**Figure 3:**
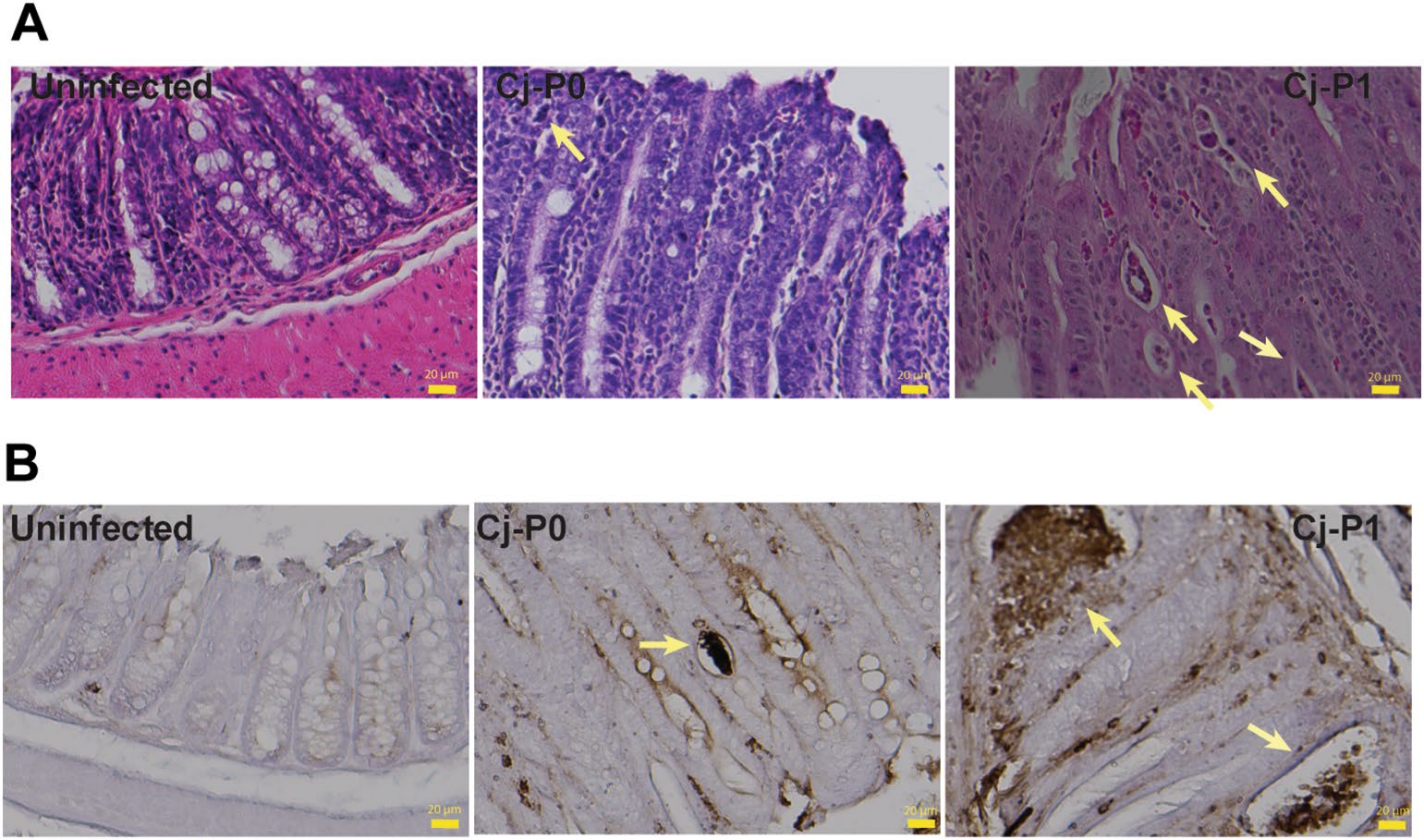
Cj-P1 strongly induced neutrophil infiltration. Cohorts of mice were infected as in Fig. 1. (**A**) Representative of intestinal high magnification histopathology of *C. jejuni*-induced colitis in *Il10*^*−/−*^ mice. (**B**) Immunohistochemistry of myeloperoxidase positive neutrophils (brown dots). Yellow arrows indicate neutrophil accumulation in the crypt lumen and formation of crypt abscesses. *P < 0.05. Data represent means ± SEM. Scale bar is 20 μm. Results are representative of 3 independent experiments.

Since *C. jejuni* infection induced strong intestinal inflammation in *Il10*^*−/−*^ mice, we examined the histopathology of the infected mice at higher magnification to have more detailed assessment. Notably, Cj-P1 depleted the majority of goblet cells and induced massive immune cell infiltration into lamina propria and numerous crypt abscesses compared to Cj-P0 (Fig. 3A). Since crypt abscesses were observed in histopathology slides, we then detected the neutrophils by targeting neutrophil marker myeloperoxidase (MPO) using immunohistopathology (IHC). As showed in Fig. 3B, Cj-P0 induced a few MPO positive cells into crypt and formed fewer crypt abscesses, whereas Cj-P1 showed stronger induction of crypt abscesses compared to Cj-P0. These results indicate that Cj-P1 gains virulence capacity to induce more infiltration of immune cells such as neutrophils.

### Transmitted Cj-P1 induced stronger migration of immune cells in vitro

**Figure 4:**
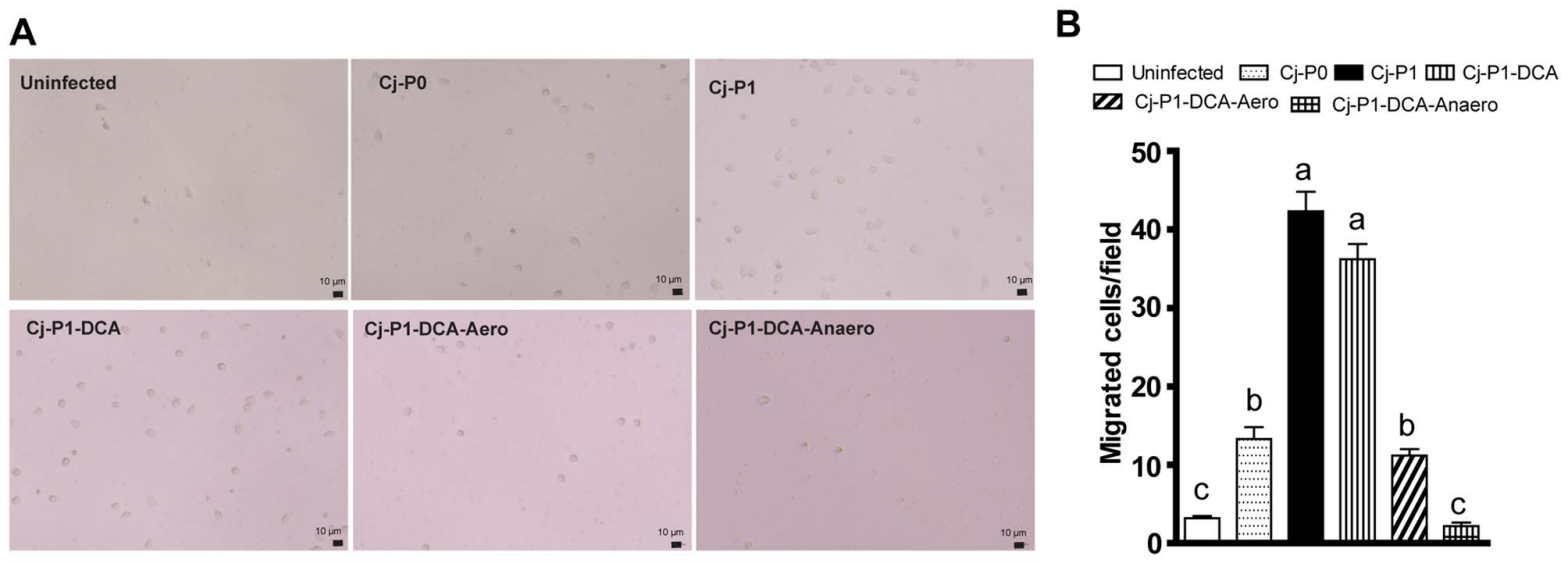
Cj-P1 strongly induced immune cell migration compared to Cj-P1-DCA-Anaero. Peripheral white blood cells were isolated and plated in a Transwell system, and the cells’ migration in response to *C. jejuni* at the bottom well was visualized and enumerated. Cells in six fields per well were counted. (**A**) Representative light images of white blood cells migrated into bottom wells. Scale bar is 10 μm. (**B**) Quantitative measurements of migrated white blood cells. Different letter of a-b means p < 0.05. Data represent means ± SEM. Results are representative of 3 independent experiments.

To understand how Cj-P1 induced more neutrophil infiltration into crypts compared to Cj-P0, we ran an in vitro immune cell migration assay similar to what we have described before^25^. Furthermore, we have reported that DCA and DCA modulated anaerobes (DCA-Anaero) prevented *C. jejuni* AR101 cecal colonization in chickens^21^, so we also isolated *C. jejuni* strains transmitted through chickens treated with DCA (Cj-P1-DCA), DCA modulated aerobe (Cj-P1-DCA-Aero), and DCA modulated anaerobe (Cj-P1-DCA-Anaero). Mouse white blood cells at 10^4^ cells/well were placed on the top inserts and Cj-P0, Cj-P1, Cj-P1-DCA, Cj-P1-DCA-Aero, and Cj-P1-DCA-Anaero were inoculated in the well bottom. One hour after the inoculation of *C. jejuni*, white blood cells migrated into the well bottom were imaged and counted under microscope. Interestingly, without *C. jejuni* infection, only a few immune cells were observed at the bottom of the wells (Fig. 4A). Cj-P1 induced the strongest immune cell migrations. Notably, Cj-P1-DCA-Anaero and Cj-P1-DCA-Aero induced 95% and 71% less immune cell migration compare to Cj-P1 (Fig. 4A,B), while Cj-P1-DCA induced comparable immune cell migration. We also examined the chicken-transmitted *C. jejuni* motility. Notably, after passaging chicken for one time, Cj-P1 increased motility by 1.36 folds compared to Cj-P0, while dietary DCA increased comparable motility compared to Cj-P1 (Supple Fig. 1). Interestingly, Cj-P1-DCA-Anaero or Cj-P1-DCA-Aero decreased the motility by 38 and 56%, respectively, compared to Cj-P1. The results suggest that *C. jejuni* transmitted through chickens increases its virulence of motility and attraction of immune cell migration, while the DCA-Anaero and DCA-Aero reduce the virulence.

### DCA-Anaero attenuated chicken transmitted Cj-P1 induction of campylobacteriosis

**Figure 5:**
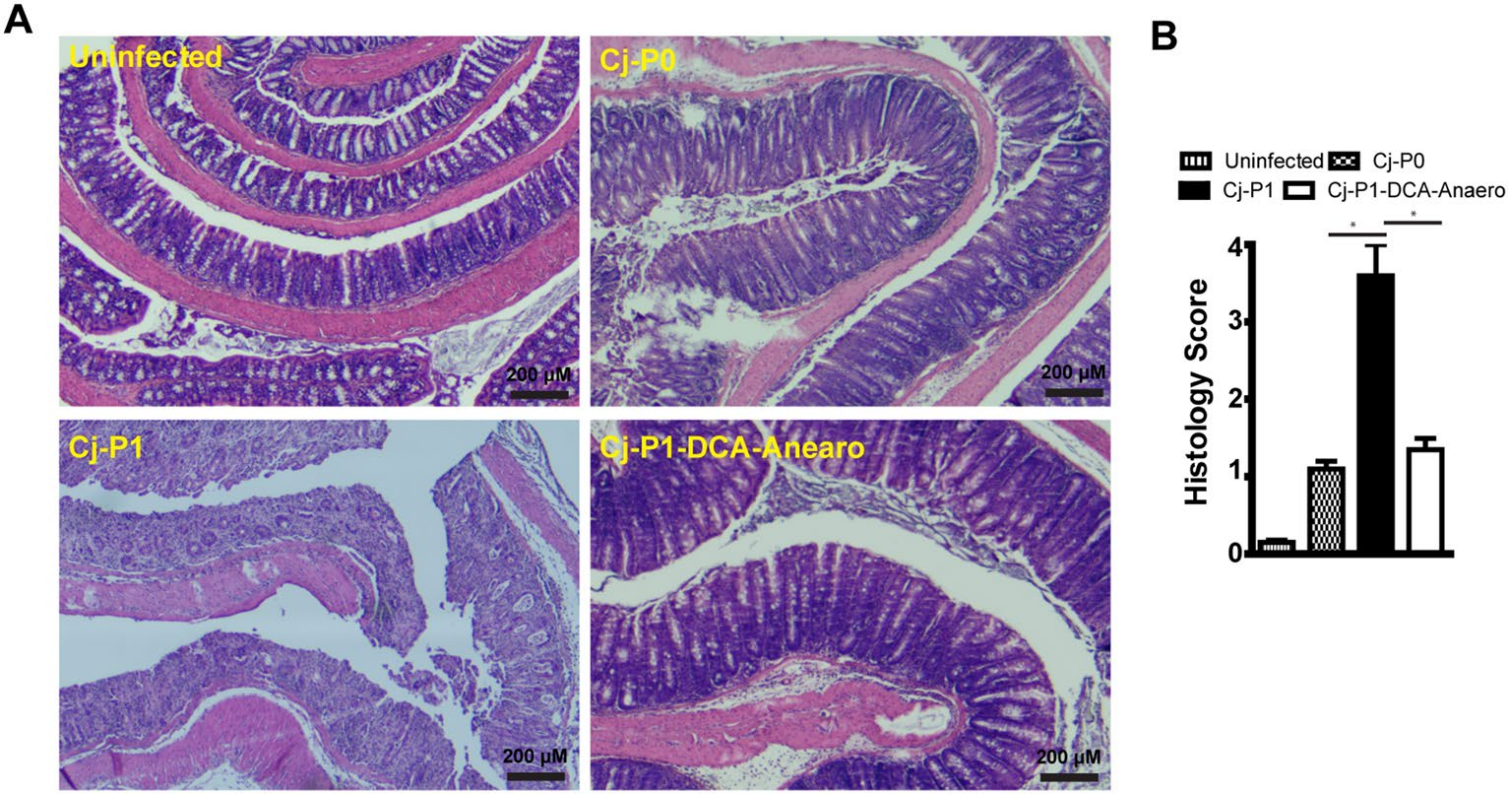
Cj-P1-DCA-Anaero induced less colitis compared to Cj-P1. Cohorts of 5–9 SPF *Il10*^*−/−*^ mice were orally gavaged daily with clindamycin for 7 days. The mice were then infected with a single dose of 10^9^ CFU /mouse *C. jejuni* of Cj-P0 (5 mice), Cj-P1 (9 mice), or Cj-P1-DCA-Anaero (5 mice) and were euthanized 8 days post-infection. (**A**) H&E staining showing representative intestinal histology of *C. jejuni*-induced colitis in *Il10*^*−/−*^ mice. (**B**) Quantification of histological intestinal damage score. Scale bar is 200 μm. *P < 0.05. Data represent means ± SEM. Results are representative of 3 independent experiments.

We then reasoned that Cj-P1-DCA-Anaero might induce less colitis in *Il10*^*−/−*^ mice compared to Cj-P1. To examine this possibility, we ran another mouse infection experiment. SPF *Il10*^*−/−*^ mice were orally gavaged daily with clindamycin for 7 days and then infected with Cj-P0, Cj-P1, and Cj-P1-DCA-Anaero. After an 8-day infection, in consistent with the previous observation, Cj-P1 induced more severe intestinal inflammation in *Il10*^*−/−*^ mice compared to Cj-P0, showed as numerous crypt abscesses, extensive immune cell infiltration, and massive goblet depletion (Fig. 5A). Remarkably, Cj-P1-DCA-Anaero induced less intestinal inflammation and histopathological score compared to Cj-P1 (Fig. 5A,B). We also infected *Il10*^*−/−*^ mice with Cj-P0, Cj-P1, Cj-P1-DCA, and Cj-P1-DCA-Aero. In line with the in vitro immune cell migration assay, comparable intestinal inflammation was induced in *Il10*^*−/−*^ mice infected with Cj-P1 and Cj-P1-DCA (Supple Fig. 2). Surprisingly, Cj-P1-DCA-Aero also induced comparable colitis to Cj-P1, although Cj-P1-DCA-Aero induced fewer white blood cell migration in vitro.

### DCA-Anaero attenuated chicken transmitted Cj-P1 invasion

**Figure 6:**
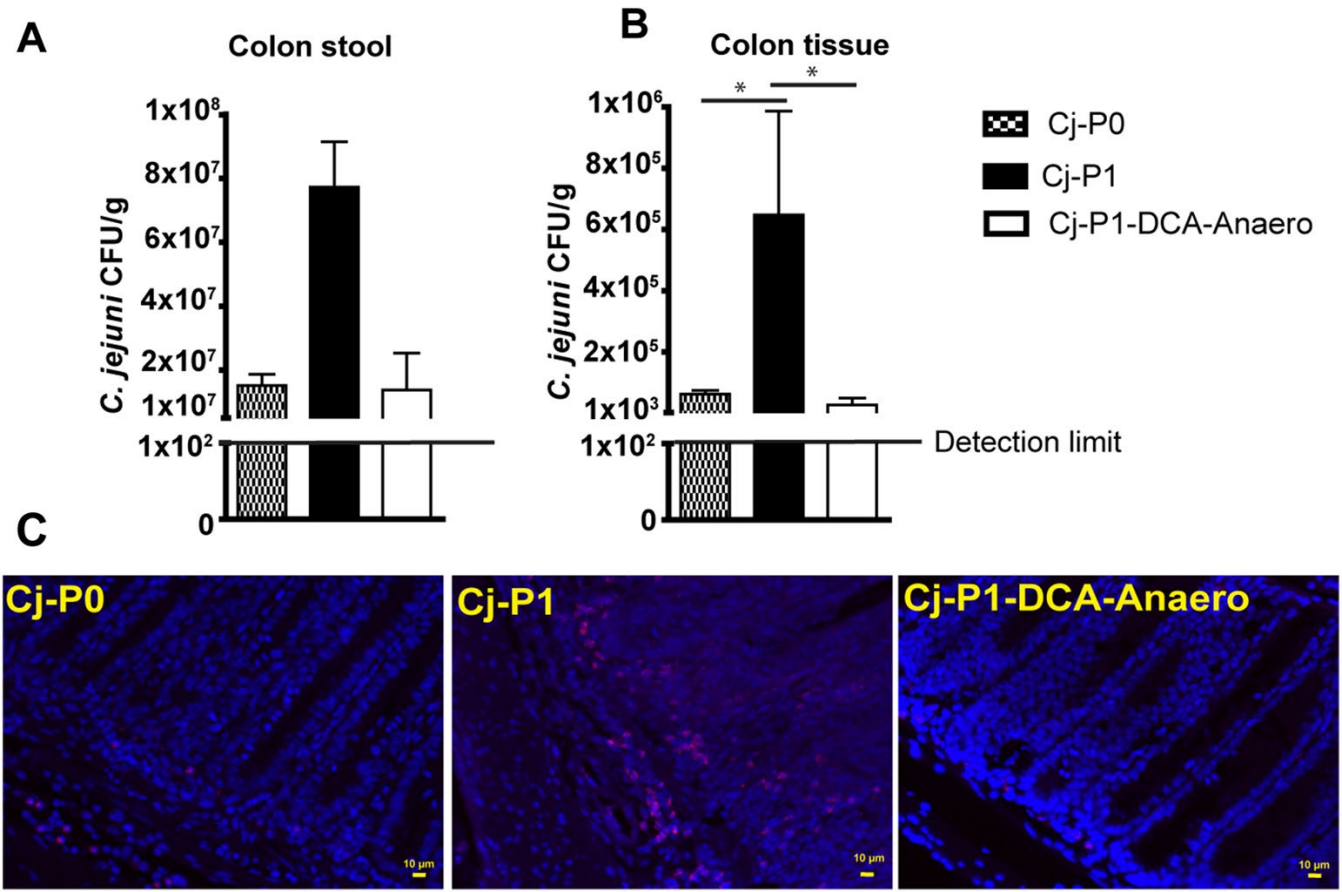
Cj-P1-DCA-Anaero colonized and invaded less compared to Cj-P1. Cohorts of mice were infected as in Fig. 5. Samples from colon content and tissue were collected and aseptically cultured. (**A**) *C. jejuni* count in colon content. (**B**) *C. jejuni* count in colon tissue. (**C**) Clustered *C. jejuni* (red dots) in colonic section of infected mice was detected using FISH. *, P < 0.05. Data represent means ± SEM. Scale bar is 10 μm. Results are representative of 3 independent experiments.

To investigate why Cj-P1-DCA-Anaero induced less intestinal inflammation, we then evaluated *C. jejuni* colonization and invasion in colon. In consistent with previous results, Cj-P1 colonized in colon was in the trend but not significant compared to Cj-P0 and Cj-P1-DCA-Anaero (Fig. 6A). Notably, Cj-P1 invades more than 100 folds in colon compared to Cj-P0, an effect attenuated by 99% in Cj-P1-DCA-Anaero (Fig. 6B). Further visualization of *C. jejuni* spatial invasion by FISH showed that Cj-P1 DNA was detected deeply in the inflamed crypts and the lamina propria, while Cj-P1-DCA-Anaero was mostly absent in the mouse colon (Fig. 6C). These results suggest that DCA-Anaero attenuates *C. jejuni* transmission-increased virulence on invasion.

### DCA-Anaero attenuated chicken transmitted Cj-P1 induction of inflammatory response

**Figure 7:**
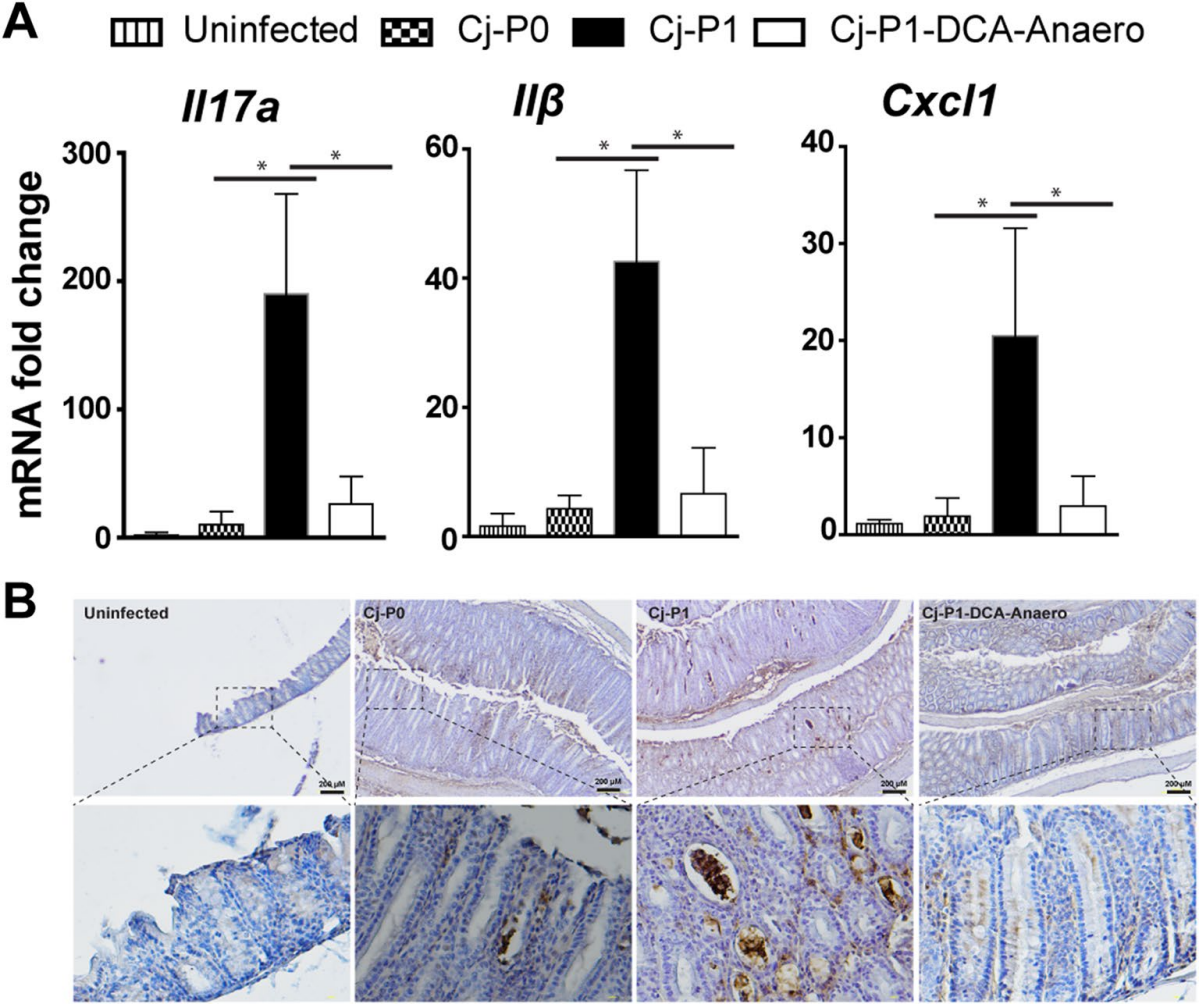
Cj-P1-DCA-Anaero induced less inflammatory response compared to Cj-P1. Cohorts of mice were infected as in Fig. 5. (**A**) *Il17a*, *Il1β*, and *Cxcl1* mRNA accumulation was quantified using real-time PCR. (**B**) IHC representation of MPO-positive cells (brown dots) in the colon tissue of *C. jejuni*-infected mice. Lower panels are magnified images of the area shown in the upper panels (scale bar, 200 μm). *P < 0.05. Data represent means ± SEM. Scale bar is 200 μm. Results are representative of 3 independent experiments.

Because of the reduced bacterial invasion and colitis in Cj-P1-DCA-Anaero compared to Cj-P1, we reasoned that the former strains would induce fewer inflammatory responses. To examine this possibility, colon tissue was collected, and RNA was extracted. Gene expression of inflammatory cytokines and chemokines were measured using real-time PCR. Cj-P0 induced inflammatory genes of *Il17a*, *Il1β*, and *Cxcl1* at 10, 4, and 2 folds, respectively, compared to uninfected mice (Fig. 7A). Remarkably, Cj-P1 induced the gene expression by 19, 10, and 11 folds, respectively, compared to Cj-P0, which was attenuated by 86, 74, and 86%, respectively, by Cj-P1-DCA-Anaero. Since the three pro-inflammatory cytokines mediated immune cell activity, we then visualized inflammatory neutrophil distribution in colon by IHC of MPO. Notably, Cj-P1 induced strong infiltration of MPO positive neutrophil and crypt abscesses, whereas Cj-P1-DCA-Anaero barely elicited neutrophil migration and crypt abscesses (Fig. 7B). These results suggest that DCA-Anaero attenuates *C. jejuni* transmission-increased virulence on the induction of inflammatory responses.

## Discussion

Although *C. jejuni* is a prevalent foodborne pathogen mainly transmitted from chickens, few approaches available to control the bacterial chicken colonization. Moreover, the microbiota and cellular events responsible for host resistance or susceptibility to *C. jejuni* infection remain largely elusive^27,28^. Foodborne *C. jejuni* is mainly monitored through enumerating the bacteria in food such as chickens. The limitation of the practice is the lack of understanding of the virulence change after chicken colonization/transmission. In previous studies, we infected chickens with *C. jejuni* AR101 (labeled as Cj-P0) and the chickens didn’t show any clinical signs and grew comparably to uninfected birds^21^. The result was consistent with previous researches that *C. jejuni* asymptomatically colonizes chickens as a commensal-like pathogen^29^. Interestingly, *C. jejuni* passaging through chickens increases colonization potential in chickens^30^ and mice^31^, however, it hasn’t been well studied if the asymptomatic transmission in chickens influenced *C. jejuni* induction of intestinal inflammation in susceptible hosts such as human or *Il10*^*−/−*^ mice. In this study, we investigated how *C. jejuni* transmitted from birds raised in different husbandry influenced its virulence in subsequent infection using *Il10*^*−/−*^ mice. The results reveal new insights regarding *C. jejuni* chicken transmission, pathogen virulence, and husbandry conditions.

One of the notable findings in this study was that chicken-transmitted *C. jejuni* (Cj-P1) increased virulence to induce stronger campylobacteriosis in mice, although chickens colonized with *C. jejuni* were healthy, shown with comparable body weight gain between uninfected and infected birds^21^. *Campylobacter jejuni* colonizes 95% flock of 20,000 chickens within 7 days after the initial one bird infected with the bacterium^32^. Transmitted *C. jejuni* increases phase-variable controlled flagellar^33^, which may contribute to its fast horizontal transmission rate in the flock. Furthermore, *C. jejuni* passaging through chicken reservoir promotes phase variation in specific contingency genes, and the populations with the variations colonize mice^31^. In consistent with these previous reports, in this study, the chicken-transmitted *C. jejuni* colonized mice with more number than the original pathogen Cj-P0. A new observation in this study is that beyond colonization ability increase, the chicken-transmitted *C. jejuni* also increased virulence to induce more severe colitis. At the host cellular level, the transmitted *C. jejuni* induced more immune cell infiltration in colon and increased immune cell migration in in vitro assay. Immune cell migration is one of the important steps in inducing campylobacteriosis^25^. Innate immune cells can directly sense bacterial cellular molecules with various receptors such as Toll-like receptors (TLR) and Nod-like receptors (NLR)^34^. It would be helpful to investigate in the future whether the chicken-transmitted *C. jejuni* had phase variations in cell surface virulence genes, such as lipooligosaccharide. In addition, it remains to be determined whether the chicken-transmitted *C. jejuni* increased virulence genes on mobility, growth, and toxin production. Together, these findings suggest that an equal count of *C. jejuni* may induce quite a different campylobacteriosis, and monitoring chicken-transmitted *C. jejuni* virulence is important for preventing foodborne campylobacteriosis.

The remarkable finding in this study is that DCA-modulated anaerobes in chickens reduced the transmitted *C. jejuni* virulence on inducing campylobacteriosis in mice (Cj-P1-DCA-Anaero vs. Cj-P1). DCA-modulated anaerobes reduce *C. jejuni* chicken colonization^21^. Conventionalized anaerobic microbiota reduces *C. jejuni*-induced intestinal inflammation in gnotobiotic mice^20^. It is well documented that microbiota prevents intestinal pathogen colonization through competitive exclusion such as virulence expression^35^, nutrition exclusion^36^, altered pH^37^, and bactericidal products^38^. However, it remains largely elusive whether microbiota influences co-inhabited pathogens on their-transmitted infection capacity. Here, we showed that DCA-modulated anaerobes in chickens reduced the transmitted *C jejuni* colonization and invasion in the large intestine of mice. Mechanistically, DCA-modulated anaerobes reduced transmitted *C jejuni* induction of immune cell migration in vitro and into the intestine as well as decreased the bacterial induction of proinflammatory mediator expression. It will be important to conduct future research on which *C. jejuni* virulence factors were down-regulated by DCA-Anaero and were responsible for the reduction of campylobacteriosis. These findings suggest that the increased chicken-transmitted *C. jejuni* virulence is feasible to be controlled with select microbiota (e.g. DCA-modulated anaerobes).

Interestingly, DCA-modulated *C. jejuni* (Cj-P1-DCA) failed to reduce intestinal inflammation, while DCA in the diet reduces *C. jejuni* chicken colonization^21^. *Campylobacter jejuni* multidrug efflux transporter gene, CmeABC, is significantly up-regulated during the initial exposure to bile acids such as DCA^39^. In consistent with the elevated production of CmeABC, bile salts in culture media promote *C. jejuni* resistance to multiple antimicrobials^39^. DCA induces *C. jejuni* virulence gene expression and invasion into epithelial cell in vitro^22^. DCA failed to reduce *C. jejuni *in vitro growth^21^. Consistently, Cj-P1-DCA showed similar motility and induction of immune cell migration compared to Cj-P1, suggesting comparable virulence on activating immune cells.

Taken together, our data revealed that DCA-modulated anaerobic microbiota not only reduced *C. jejuni* colonization in chickens, but also reduced chicken-transmitted campylobacteriosis. The reduction of virulence reflected on immune cell migration and infiltration into the intestine, *C. jejuni* invasion, pro-inflammatory response, and collectively intestinal inflammation. These findings highlighted the importance to monitor chicken-transmitted *C. jejuni* virulence in addition to colonization counts. Although it is a new concept, using select microbiota in poultry production is a key step to successfully prevent foodborne campylobacteriosis.

## Supplementary information

Supplementary Information.

Supplementary Figure 1.

Supplementary Figure 2.

## Data Availability

Data sharing is not applicable to this article as no datasets were generated or analyzed during the current study.
