# Supplementary material for: Microbiota attenuates chicken transmission-exacerbated campylobacteriosis in Il10−/− mice
Source: Sci Rep. 2020 Nov 30;10:20841. doi: 10.1038/s41598-020-77789-2 (PMC7705718; doi:10.1038/s41598-020-77789-2)
Supplement: Supplementary file 1 — Supplementary Information. [file 41598_2020_77789_MOESM1_ESM.docx]

**Microbiota attenuates chicken transmission-exacerbated campylobacteriosis in *Il10^-/-^* mice**

***Short Title*: Microbiota reduces transmitted campylobacteriosis**

Ying Fu*^,#^, Ayidh Almansour*^,#^, Mohit Bansal^#^, Tahrir Alenezi*^,#^, Bilal Alrubaye*^,#^, Hong Wang^#^, and Xiaolun Sun^#,^*^, $^

*CEMB, ^#^Center of Excellence for Poultry Science, University of Arkansas, Fayetteville AR 72701

**Supplemental Figure 1. Passaging *C. jejuni* in chickens increased its motility.** Cj-P0, Cj-P1, Cj-P1-DCA, Cj-P1-DCA-Anaero or Cj-P1-DCA-Aero at 1 μl was stabbed into a 0.4% agar Brain Heart Infusion (BHI) plate without antibiotic cocktail. Following microaerobic growth at 42°C for 24 h, the radius of the ring was calculated relative to that of Cj-P0. Experiments were performed in triplicate and repeated three times. *, P < 0.05. Results are representative of 3 independent experiments.

**Supplemental Figure 2. Cj-P1-DCA and Cj-P1-DCA-Aero induced comparable campylobacteriosis to Cj-P1.** Cohorts of 5-9 SPF *Il10^-/-^* mice were orally gavaged daily with clindamycin for 7 days. The mice were then infected with a single dose of 10^9^ CFU /mouse *C. jejuni* of Cj-P0 (5 mice), Cj-P1 (9 mice), Cj-P1-DCA (9 mice), or Cj-P1-DCA-Aero (5 mice) and were euthanized 8 days post-infection. (A) H&E staining showing representative intestinal histology of *C. jejuni*-induced colitis in *Il10^-/-^* mice. (B) Quantification of histological intesti­nal damage score. *, P<0.05. Scale bar is 200 μm. Results are representative of 3 independent experiments.
