# Supplementary figures and images for: Microbiota attenuates chicken transmission-exacerbated campylobacteriosis in Il10−/− mice
Source: Sci Rep. 2020 Nov 30;10:20841. doi: 10.1038/s41598-020-77789-2 (PMC7705718; doi:10.1038/s41598-020-77789-2)

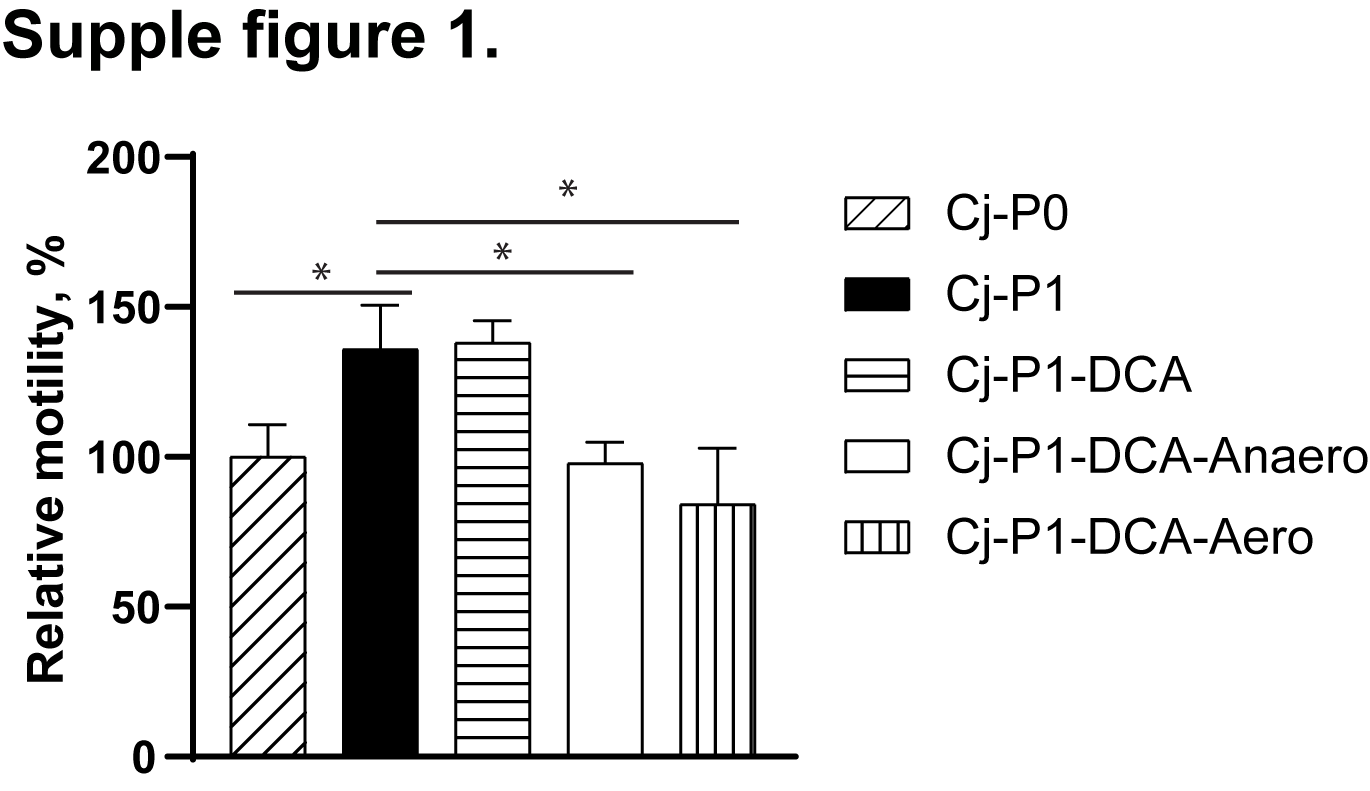

Supplement: Supplementary file 2 — Supplementary Figure 1. [file 41598_2020_77789_MOESM2_ESM.tif]

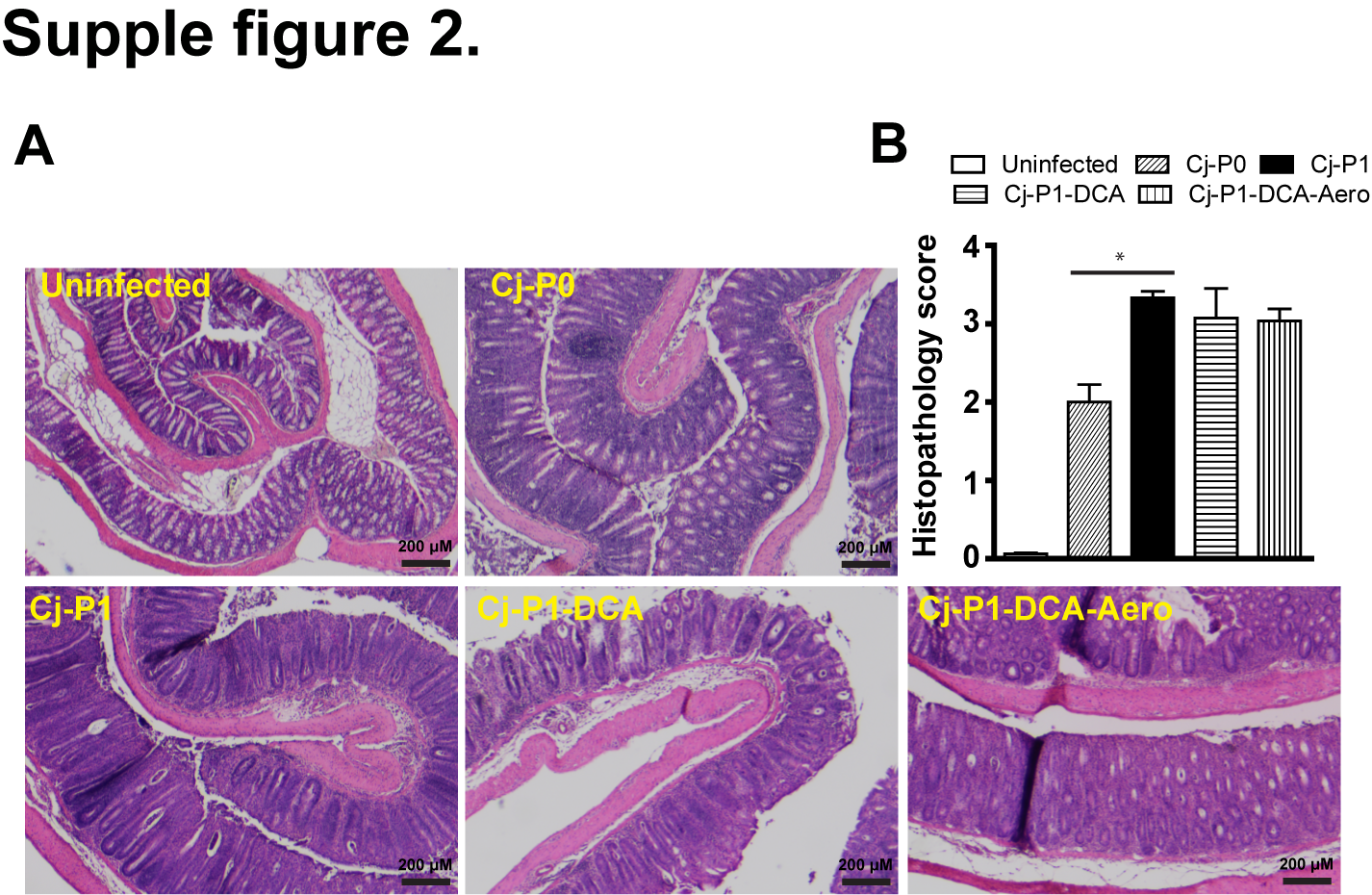

Supplement: Supplementary file 3 — Supplementary Figure 2. [file 41598_2020_77789_MOESM3_ESM.tif]
